# Antifungal Treatment for Pityriasis Versicolor

**DOI:** 10.3390/jof1010013

**Published:** 2015-03-12

**Authors:** Aditya K. Gupta, Kelly A. Foley

**Affiliations:** 1 Department of Medicine, University of Toronto, Toronto, ON M5G 2C4, Canada; 2 Mediprobe Research Inc., London, ON N5X 2P1, Canada; E-Mail: kfoley@mediproberesearch.com

**Keywords:** tinea versicolor, *Malassezia*, topical antifungals, oral antifungals, ketoconazole

## Abstract

Background: Pityriasis versicolor (PV), also known as tinea versicolor, is caused by *Malassezia* species. This condition is one of the most common superficial fungal infections worldwide, particularly in tropical climates. PV is difficult to cure and the chances for relapse or recurrent infections are high due to the presence of *Malassezia* in the normal skin flora. This review focuses on the clinical evidence supporting the efficacy of antifungal treatment for PV. Method: A systematic review of literature from the PubMed database was conducted up to 30 September 2014. The search criteria were “(pityriasis versicolor OR tinea versicolor) AND treatment”, with full text available and English language required. Conclusions: Topical antifungal medications are the first-line treatment for PV, including zinc pyrithione, ketoconazole, and terbinafine. In cases of severe or recalcitrant PV, the oral antifungal medications itraconazole and fluconazole may be more appropriate, with pramiconazole a possible future option. Oral terbinafine is not effective in treating PV and oral ketoconazole should no longer be prescribed. Maintenance, or prophylactic, therapy may be useful in preventing recurrent infection; however, at this time, there is limited research evaluating the efficacy of prophylactic antifungal treatment.

## 1. Introduction

Pityriasis versicolor (PV) is a chronic cutaneous fungal infection caused by proliferation of lipophilic yeast (*Malassezia* species) in the stratum corneum [1,2]. The most common Malassezia species associated with PV is *M. globosa*, with *M. sympodialis* and *M. furfur* also frequently seen [3]. In most cases of PV, *Malassezia*, as a part of normal skin flora, are not pathogenic unless they assume a mycelial form [2]. This may be triggered by various factors, including humidity and high temperature, hyperhidrosis, familial susceptibility, and immunosuppression [1,2]. Consequently, PV occurs more frequently in tropical climates (as much as 40%) as compared to temperate climates [3]. PV is difficult to cure, as relapse following treatment can be as high as 80% within 2 years [4].

Patients with PV present with well demarcated round or oval macules on the trunk, neck, and upper arms where the density of sebaceous glands is high. These lesions often appear hyperpigmented on lighter skin types and hypopigmented in darker or tanned skin and can vary in color [5]. Smaller macules may have a powdery appearance due to flaking skin, although flaking may only manifest on the edges of larger lesions [2]. PV is generally asymptomatic, although some patients experience mild pruritus. By far, the greatest concern for patients lending to their seeking treatment is the unpleasant cosmetic appearance of the skin [2]. Unfortunately, altered pigmentation can persist following treatment. This is not often used as a criterion for treatment efficacy, with mycological cure (negative microscopy) and alleviation of physical symptoms such as lesion clearance, erythema, pruritus, and desquamation preferred.

Diagnosis of PV is confirmed by microscopy using skin scrapings from the borders of lesions, or, if this is not possible, obtaining samples using the transparent tape method. Wood’s light examination may also aid in diagnosis, with lesions appearing yellow or gold [2,6]. Topical antifungals are currently the first line of treatment for PV and systemic antifungals are recommended for severe or recalcitrant cases [7]. There are, however, many non-specific topical treatments that may be effective in treating PV [8,9]. In some cases, misdiagnosis may lead to inappropriate and ineffective treatment (e.g., antibiotics, corticosteroids) [5]. The focus of the present review is to highlight the clinical evidence supporting the use of topical and systemic antifungal medications in treating PV.

## 2. Topical Treatment for Pityriasis Versicolor

Effective topical treatment for PV includes creams, lotions, and shampoos. These are applied daily or twice daily for varying periods of time, quickly improving clinical symptoms. Patient compliance may be affected by multiple, laborious applications, or minor skin irritation. Non-specific topical treatments for PV do not act specifically against *Malassezia* species. Rather, they physically or chemically remove dead infected tissue [2]. Non-specific treatments shown to be effective in treating PV include selenium sulphide (lotion, cream, or shampoo), zinc pyrithione, propylene glycol, and Whitfield’s ointment [8,9].

There are multiple topical medications, such as bifonazole, clotrimazole, and miconazole, that have direct fungistatic activity and are shown to be effective in treating PV (for an extensive review, see Gupta *et al.*, 2005 [9]). In many cases, these and non-specific agents are used in studies to demonstrate the comparable efficacy of the newer topical and oral antifungals [10,11,12,13]. For example, twice daily application of ciclopirox olamine cream 1% for 14 days was significantly more effective than 1% clotrimazole cream (mycological cure 77% *vs.* 45%, *p* ≤ 0.001) [14]. While evidence suggests that non-specific agents and older azoles can be effective in treating PV [7,8,9,10,11,12,13], the topical antifungals most extensively investigated recently are ketoconazole (Table 1) and terbinafine (Table 2).

**Table 1:** Clinical studies evaluating the efficacy of topical ketoconazole.

Reference	Design	Treatment Regimen	No.	Mycological Cure	Complete Cure	Follow-Up (Cure or Relapse)
Savin *et al.* 1986 [15]	DB, R	2% ketoconazole cream, 1×/day for 14 days	51	43/51 = 84% ***	43/51 = 84% ***	Cure rate: 38/48 = 79% (12 months)
		Placebo cream	50	11/50 = 22%	5/50 = 10%	16/48 = 33% (24 months)
Balwada *et al.* 1996 [12]	DB, R	2% ketoconazole cream, 1×/day for 14 days	20	18/20 = 90%	18/20 = 90%	Cure rate: 16/16 = 100% (8 weeks)
		1% clotrimazole cream	20	17/20 = 85%	16/20 = 80%	16/16 = 100% (8 weeks)
Chopra *et al.* 2000 [16]	R	2% ketoconazole cream, 1×/day for 14 days	25	22/25 = 88%	20/25 = 80%	Relapse: 3 patients (3 months)
		1% terbinafine cream	25	24/25 = 96%	24/25 = 96%	2 patients (3 months)
Lange *et al.* 1998 [17]	DB, R	2% ketoconazole shampoo, 1×/day, for 3 days	106	At Day 31, 89/106 = 84% **	At Day 31, 77/106 = 73% **	-
		2% ketoconazole shampoo, 1 day, placebo days 2, 3	103	79/101 = 78% **	71/103 = 69% **	-
		Placebo 1×/day, for 3 days	103	11/103 = 11%	5/103 = 5%	-
Aggarwal *et al.* 2003 [11]	R	2% ketoconazole shampoo, 1×/week for 3 weeks	20	At 4 weeks, 19/20 = 95%	-	Relapse: 1 patient (3 months)
		2.5% selenium sulphide shampoo	20	17/20 = 85%	-	2 patients (3 months)
Rathi 2003 [18]	O	2% ketoconazole shampoo, 1×/day, for 3 days	30	At Day 31, 27/30 = 90%	-	-
Rigopoulos *et al.* 2007 [19]	DB, R	2% ketoconazole shampoo, 1×/day for 14 days	26	At Day 28, 21/26 = 81%	At Day 28, 21/26 = 81%	-
		1% flutrimazole shampoo	29	22/29 = 76%	22/29 = 76%	-
Di Fonzo *et al.* 2008 [20]	R	1% ketoconazole foam, 1×/day for 14 days	22	At 5 weeks, 18/18 = 100%	At 5 weeks, 5/18 = 28%	Complete cure rate: 9/11 = 82% (3 months)
		2% ketoconazole cream	24	19/19 = 100%	9/19 = 47%	12/13 = 92% (3 months)
Cantrell *et al.* 2014 [21]	O	2% ketoconazole foam, 2×/day for 14 days	11	At 4 weeks, 6/11 = 55%	-	Relapse: 1 patient (4 weeks)
Shi *et al.* 2014 [22]	DB, R	2% ketoconazole cream + 0.1% adapalene gel, 1×/day for 14 days	50	At 4 weeks, 46/50 = 92% **	-	-
		2% ketoconazole cream, 2×/day for 14 days	50	At 4 weeks, 36/50 = 72%	-	-

** *p* < 0.01; *** *p* < 0.001—Treatment significantly different from placebo. DB: double-blind; O: open; R: randomized.

**Table 2:** Clinical studies evaluating the efficacy of topical terbinafine.

Reference	Design	Treatment Regimen	No.	Mycological Cure	Complete Cure	Follow-Up (Cure or Relapse)
Kagawa 1989 [23]	O	1% terbinafine cream, 2×/day for 14 days	87	78/87 = 90%	-	-
Aste *et al.* 1991 [10]	SB, R	1% terbinafine cream, 2×/day up to 4 weeks	20	At 4 weeks, 20/20 = 100%	At 4 weeks, 20/20 = 100%	-
		1% bifonazole cream	20	19/20 = 95%	19/20 = 95%	-
Faergemann *et al.* 1997 [24]	DB, R	1% terbinafine emulsion gel, 1×/day, for 7 days	28	At 8 weeks, 21/28 = 75% ***	At 8 weeks, 21/28 = 75% ***	-
		Placebo gel	29	4/29 = 14%	4/29 = 14%	-
Vermeer *et al.* 1997 [25]	DB, R	1% terbinafine solution, 2×/day for 7 days	76	At 8 weeks, 62/76 = 81% ***	At 8 weeks, 36/76 = 47%	-
		Placebo	34	14/34 = 41%	10/34 = 29%	-
Savin *et al.* 1999 [26]	DB, R	1% terbinafine solution, 2×/day for 7 days	102	46/96 = 48% *	-	Myc. cure rate: 69/85 = 81% * (8 weeks)
		Placebo solution	50	14/46 = 30%	-	13/43 = 30% (8 weeks)
Budimulja *et al.* 2002 [27]	DB, R	1% terbinafine solution, 2×/day for 7 days	192	At 2 weeks, 108/192 = 56% ***	-	Relapse (from week 4 to 8): 2 patients Myc. cure rate (8 weeks):123/192 = 64% ***
		Placebo	96	34/96 = 35%	-	32/96 = 33% (8 weeks)
Budimulja *et al.* 2002 [27]	DB, R	1% terbinafine solution, 1×/day for 7 days	50	At 1 week, 37% *	-	Myc. cure rate: 49% * (8 weeks)
		Placebo	50	17%	-	27% (8 weeks)

* *p* < 0.05; *** *p* < 0.001—Treatment significantly different from placebo. DB: double-blind; O: open; R: randomized; SB: single-blind.

### 2.1. Ketoconazole

Ketoconazole, an imidazole, was the first broad-spectrum antifungal used in the treatment of superficial and systemic mycoses. Through inhibition of the enzyme lanosterol 14α-demethylase, ketoconazole disrupts ergosterol biosynthesis to limit cell function and growth [28]. Multiple formulations have proved effective in treating PV, including cream, shampoo, and foam (Table 1), with the most common regimen being once daily application of cream or foam for 14 days. Ketoconazole cream has been shown to be as effective as 1% clotrimazole [12] and 1% terbinafine cream [16], whereas ketoconazole shampoo was shown to be as effective as 2.5% selenium sulphide [11] and 1% flutrimazole shampoo [19].

Application of ketoconazole shampoo has varied across studies, including once daily for 3- [17,18] or 14 days [19], and once weekly for 3 weeks [11]. Lange *et al.* (1998) conducted a multi-center, double-blind, randomized, placebo-controlled clinical trial evaluating the efficacy of a single application of ketoconazole shampoo *vs.* daily application for 3 days [17]. Patients used ketoconazole shampoo either daily for 3 days, ketoconazole once followed by placebo shampoo for 2 days, or placebo shampoo for 3 days. Thirty-one days from the start of treatment, there were no significant differences between the two ketoconazole regimens in mycological or complete cure rates. Both ketoconazole regimens, daily application for 3 days and one application, were significantly more effective than placebo shampoo for mycological cure (84% *vs.* 78% *vs.* 11% respectively, *p* < 0.001) and complete cure (73% *vs.* 69% *vs.* 5% respectively, *p* < 0.001) [17].

In studies that followed patients well beyond the treatment period (3–24 months), relapse and/or lower cure rates were observed [11,15,16,21]. However, ketoconazole foam or cream applied once daily for 14 days appear to have some ability in maintaining complete cure 3–12 months post-treatment [15,20]. Seventy nine percent of patients displayed complete cure at 12 months post-treatment with 2% ketoconazole cream [15], while 82% and 92% of patients displayed complete cure measured 3 months post-treatment with ketoconazole 1% foam and 2% cream, respectively [20]. Potential advantages to using 1% ketoconazole foam include a shorter evaporation time, and increased transcutaneous penetration for a longer time in the epidermis compared to creams or lotions [20,21].

Recently, a combination of ketoconazole cream with 0.1% adapalene gel was compared to ketoconazole cream alone in a double blind, randomized clinical trial [22]. Adapalene gel is a derivative of naphthoic acid used to treat acne that acts by inhibiting cellular differentiation [29]. Previously, twice daily application of ketoconazole 2% cream for 14 days was shown to be equivalent to 0.1% adapalene gel in treating PV [30]. In the latest study, patients applied either a combination of 2% ketoconazole cream and 0.1% adapalene gel once daily for 14 days or 2% ketoconazole cream twice daily for 14 days. Combination treatment produced clinical improvement and mycological cure faster (within 2 weeks) than monotherapy. By week 4, the treatment with the combination of ketoconazole and adapalene produced significantly greater clinical improvement and mycological cure as compared to ketoconazole monotherapy (92% *vs.* 72%, *p* = 0.009) [22]. Mild adverse events were reported in treatment groups and included erythema, skin dryness, and burning sensation with the combination treatment or mild irritation with monotherapy [22]. Combination treatments may show promise for future treatment of PV. The relative efficacy of different topical ketoconazole formulations is difficult to ascertain, as cure rates at 2–4 weeks were high for all formulations.

### 2.2. Terbinafine

Terbinafine, an allylamine, exhibits fungicidal action against dermatophytes, yeasts, and molds [31]. Terbinafine acts by inhibiting squalene epoxidase, thereby blocking sterol biosynthesis and altering fungal cell membrane integrity [32]. Terbinafine cream was equivalent to topical ketoconazole and bifonazole cream, with mycological and complete cures ranging from 88% to 100% [10,16]. In addition, the mean duration of treatment (maximum 4 weeks) until mycological cure with twice daily application of 1% terbinafine cream was significantly shorter than that of twice daily 1% bifonazole cream [10].

Multiple double blind, randomized, placebo-controlled studies have investigated the efficacy of 1% terbinafine solution applied twice daily for 7 days [25,26,27]. Seven weeks following a 7-day course of twice daily terbinafine solution, both Vermeer *et al.* [25] and Savin *et al.* [26] reported a mycological cure rate of 81%, significantly greater than placebo (41%, *p* < 0.001; 30%, *p* < 0.001, respectively). When clinical effectiveness was evaluated as absence or nearly complete absence of physical symptoms combined with mycological cure, terbinafine was significantly more effective than placebo immediately following the completion of treatment (48% *vs.* 30%, *p* < 0.05) and 7 weeks later (81% *vs.* 30%, *p* < 0.001) [26]. Additionally, patient ratings of treatment efficacy were significantly higher for terbinafine *vs.* placebo (*p* < 0.001) [26].

Budimulja and Paul (2002) conducted two double blind, randomized, placebo-controlled trials of 1% terbinafine solution [27]. Both trials administered terbinafine for 7 days, with one requiring twice daily application and the other once daily application. Eight weeks following the start of treatment, twice daily application of terbinafine produced a mycological cure rate of 64% and once daily application a mycological cure rate of 49%. The tropical climate (Indonesia) of this study, where PV is difficult to treat, likely contributed to lower cure rates compared to previous studies [27]. Regardless, effective treatment of PV with twice daily terbinafine solution can be achieved (Table 2).

## 3. Oral Treatment for Pityriasis Versicolor

Oral, or systemic, antifungals are effective in treating a variety of infections, but can be associated with serious adverse events. Use of oral antifungals to treat PV are considered second line treatment and used for recalcitrant or severe infections. In the case of terbinafine, oral treatment is not effective in PV [33]. Unlike other antifungals, terbinafine is not excreted in sweat and may not reach high enough concentrations in the stratum corneum to exhibit fungicidal action against Malassezia species [34,35]. However, as mentioned previously, topical terbinafine does not have this restriction and can be effective.

Ketoconazole, once the gold standard for oral treatment of fungal infections, is no longer suggested for treatment of superficial mycoses, including PV, in Canada, the US, or Europe. The risk of hepatotoxic adverse events associated with oral ketoconazole (estimated at approximately 1 in 500) [36,37,38] was determined to outweigh potential benefits, with North American government agencies in 2013 recommending oral ketoconazole only for severe or life-threatening systemic mycoses [39,40], while in 2013, Europe and Australia withdrew oral ketoconazole from the market [41]. Newer antifungals have been shown to have similar efficacy as oral ketoconazole in treating PV [42,43,44,45]. Today, oral treatments include itraconazole (Table 3), fluconazole (Table 4), and pramiconazole (Table 5).

**Table 3:** Clinical studies evaluating the clinical efficacy of itraconazole.

Reference	Design	Treatment Regimen	No.	Mycological Cure	Complete Cure
Galimberti *et al.* 1987 [46]	O, R	200 mg for 5 days, itraconazole	13	10/13 = 77%	10/13 = 77%
		200 mg for 7 days, itraconazole	15	13/15 = 87%	13/15 = 87%
Morales-Doria 1987 [47]	O, R	200 mg for 5 days, itraconazole	24	At Day 28, 19/20 = 95%	-
		100 mg for 5 days, itraconazole	23	20/20 = 100%	-
Hickman 1996 [48]	DB, R	200 mg for 7 days, itraconazole	18	At Day 35, 16/18 = 89% **	-
		Placebo	18	1/18 = 6%	-
Ravikumar *et al.* 1999 [49]	DB, R	400 mg single dose itraconazole	12	At 8 weeks, 2/12 = 17%	-
		Placebo	13	0/13 = 0%	-
Kokturk *et al.* 2002 [50]	O, R	200 mg for 5 days, itraconazole	20	At Day 28, 14/20 = 70% **	At Day 28, 14/20 = 70% **
		400 mg single dose itraconazole	20	4/20 = 20%	4/20 = 20%
		400 mg for 3 days, itraconazole	20	15/20 = 75% **	15/20 = 75% **
Kose *et al.* 2002 [51]	O, R	400 mg single dose itraconazole	24	At 6 weeks, 85%	-
		200 mg for 7 days, itraconazole	26	90%	-
Faergemann *et al.* 2002 [52] (prophylaxis)	DB, R	400 mg, 1×/month for 6 months, itraconazole	106	At 6 months, 90/102 = 88% ***	-
		Placebo	103	56/99 = 57%	-

** *p* < 0.01; *** *p* < 0.001—Treatment significantly different from placebo or comparator. DB: double-blind; O: Open; R: Randomized.

**Table 4:** Clinical studies evaluating the efficacy of fluconazole.

Reference	Design	Treatment Regimen	No.	Mycological Cure	Complete Cure	Follow-Up (Cure or Relapse)
Kose *et al.* 1995 [53]	O	600 mg for 14 days, fluconazole	27	88%	80%	Relapse: 14% (12 weeks)
		400 mg for 14 days, itraconazole	25	80%	74%	20% (12 weeks)
Amer 1997 [54]	O, R	150 mg/week, 4 weeks fluconazole	207	At week 1, 17/207 = 8%	-	28 days after last dose, Myc. cure: 151/207 = 73%
		300 mg/week, 4 weeks fluconazole	190	29/190 = 15% ***	-	177/190 = 93%
		300 mg biweekly, fluconazole	206	69/206 = 34% ***	-	179/206 = 87%
Sankara Rao *et al.* 1997 [55]	O	400 mg single dose fluconazole	25	At 8 weeks, 23/25 = 92%	-	-
Balachandran *et al.* 1999 [56]	DB, R	400 mg single dose fluconazole	18	At 2 weeks, 8/18 = 44%	-	-
		Placebo	12	1/12 = 8%	-	-
Montero-Gei *et al.* 1999 [57]	O, R	450 mg single dose fluconazole	30	At Day 30, 21/30 = 70%	-	Relapse (Day 60) 6/29 = 21%
		300 mg/week, 2 weeks fluconazole	30	29/30 = 97% *	-	6/30 = 20%
		200 mg for 7 days, itraconazole	30	24/30 = 80%	-	1/27 = 4%
Bhogal *et al.* 2001 [45]	O, R	400 mg single dose fluconazole	45	At 4 weeks, 37/45 = 82%	-	Relapse (12 months): 0/30 = 0%
		150 mg/week, 4 weeks, fluconazole	45	29/45 = 64%	-	2/29 = 7%
		400 mg single dose ketoconazole	45	24/45 = 53%	-	6/24 = 25%
		200 mg for 10 days ketoconazole	45	33/45 = 73%	-	1/28 = 4%
Partap *et al.* 2004 [58]	O, R	400 mg single dose fluconazole	20	At 8 weeks, 13/20 = 65% *	At 8 weeks, 4/20 = 20%	Relapse (8weeks): 7/20 = 35%*
		400 mg single dose itraconazole	20	4/20 = 20% *	1/20 = 5%	12/20 = 60%
Karakas *et al.* 2005 [59]	O	300 mg/week, 2 weeks, fluconazole	44	At 4 weeks, 31/40 = 78%	At 4 weeks, 30/40 = 75%	Relapse: 0% (12 weeks)
Dehghan *et al.* 2010 [13]	DB, R	400 mg single dose fluconazole, placebo cream 2×/day, 14 days	50	-	At 4 weeks, ^a^ 41/50 = 82%	At 12 weeks, ^a^ 46/50 = 92%
		Placebo pill, 1% clotrimazole cream 2×/day, 14 days	55	-	52/55 = 95% *	45/55 = 82%

^a^ Did not measure mycological or complete cure. Values represent clinical response: ≥95% lesion clearance. * *p* < 0.05; *** *p* < 0.001—Treatment significantly different from placebo or comparator. DB: double-blind; O: open; R: randomized.

**Table 5:** Clinical studies evaluating the efficacy of pramiconazole.

Reference	Design	Treatment Regimen	No.	Mycological Cure	Complete Cure
Faergemann *et al.* 2007 [60]	O	200 mg for 3 days, pramiconazole	19	At Day 30, 19/19 = 100%	At Day 30, 19/19 = 100%
Faergemann *et al.* 2009 [61]	DB, R	100 mg single dose pramiconazole	26	At Day 28, 11/26 = 42%	At Day 28, 9/26 = 35%
		200 mg single dose pramiconazole	22	15/22 = 68% ***	13/22 = 59% **
		200 mg for 2 days, pramiconazole	25	23/25 = 92% ***	18/25 = 72% ***
		200 mg for 3 days, pramiconazole	26	25/26 = 96% ***	22/26 = 85% ***
		400 mg single dose pramiconazole	23	18/23 = 78% ***	12/23 = 52% *
		Placebo	25	4/25 = 16%	4/25 = 16%

* *p* < 0.05; ** *p* < 0.01; *** *p* < 0.001—Treatment significantly different from placebo. DB: double-blind; O: open; R: randomized.

### 3.1. Itraconazole

Itraconazole, a triazole antifungal, alters fungal cell function similarly to ketoconazole, through inhibition of cytochrome P450-dependent ergosterol synthesis [28]. To effectively treat PV, a minimum total amount of 1000 mg itraconazole over the course of treatment was required to produce a significant mycological response [51]. Treatment once daily for 5 days with 200 mg itraconazole shows high efficacy up to one month following treatment [47,50] and is recommended for treatment of PV [62]. A 7-day course of treatment is the standard regimen for itraconazole (Table 3) [46,48,52].

Studies of 5 and 7-day regimens reported that the two regimens are comparable [46,63]. Following treatment with oral itraconazole, 80% of patients treated for 5 or 7 days experienced decreased physical symptoms and negative microscopy [63]. Galimberti *et al.* (1987) showed that 7 days of itraconazole produced slightly higher cure rates than 5 days, but statistical analysis was not performed [46]. Importantly, abnormalities in fungus structure were observed immediately after completion of treatment; however, these processes were not complete until 28 days following treatment [46], emphasizing the long-term action of oral antifungals and the need to assess clinical and mycological cure well after oral treatments have been completed.

Studies have evaluated the efficacy of 400 mg itraconazole administered once and for 3 days as compared to 200 mg itraconazole for 5 or 7 days [50,51]. While Kose *et al.* (2002) demonstrated that a single 400 mg dose was equivalent to 200 mg for 7 days [51], Kokturk *et al.* (2002) found a single 400 mg dose to be ineffective, with itraconazole regimens of 400 mg for 3 days and 200 mg for 5 days both producing significantly greater mycological and complete cure (*p* = 0.001) [50]. Although a regimen of 400 mg itraconazole for 3 days may be an alternative to 200 mg itraconazole for 5 days, there is not enough evidence at this time to warrant changing recommendations from 5 day treatment.

Recurrence of PV following cessation of symptoms is typical within 6 months to 2 years after extensive treatment. As such, antifungal prophylaxis is of interest to prevent recurrence. Following an open trial of 200 mg itraconazole for 7 days with a 4 week follow-up, the 205 patients exhibiting mycological (negative microscopy) cure (205/223 = 92%) were entered into a double blind, randomized, placebo controlled trial [52]. Itraconazole was administered once per month for 6 months as relapse prophylaxis (200 mg twice a day). At the end of 6 months, 88% of patients receiving prophylactic itraconazole were still mycologically cured, while only 57% of patients receiving placebo as prophylaxis were mycologically cured (*p* < 0.001). Additionally, clinical symptoms (erythema, desquamation, itching, and hypopigmentation) were significantly fewer in prophylactic itraconazole patients (*p* < 0.001) [52].

### 3.2. Fluconazole

Fluconazole is a triazole antifungal, inhibiting cytochrome P450-dependent ergosterol synthesis similarly to itraconazole and ketoconazole [28]. Studies have shown that fluconazole is equivalent to [42,44], or more effective than [45], oral ketoconazole in treating PV. A large randomized trial conducted by Amer (1997) demonstrated the efficacy of weekly regimens of fluconazole: 150 mg or 300 mg weekly for 4 weeks, or 300 mg bi-weekly for 4 weeks [54]. Four weeks after the last treatment, mycological cure for the regimens of 300 mg fluconazole (weekly 93%, bi-weekly 87%) were significantly higher than 150 mg fluconazole (73%, *p* < 0.0001) [54]. Two weekly doses of 300 mg fluconazole is the recommended treatment for PV [63]. This regimen produced a significantly higher mycological cure rate (97%) compared to a single 450 mg dose of fluconazole (*p* = 0.012) [57] and in an open study, 12 weeks following the start of treatment, all patients who had complete and mycological cure at week 4 had not shown relapse [59].

Recently, the efficacy of a single dose of 400 mg fluconazole in treating PV has been investigated. A single dose of 400 mg fluconazole produced a significantly greater mycological cure rate than a single dose of 400 mg ketoconazole four weeks after treatment (82% *vs.* 53%, *p* < 0.01) [45]. Weekly treatment with 150 mg fluconazole for four weeks also produced a high mycological cure rate (64%) [45]. Patients were followed up 12 months after treatment to assess relapse, with 0% and 7% of patients receiving a single dose or weekly fluconazole experiencing recurring symptoms. Relapse was found in significantly more patients receiving a single dose of itraconazole compared to a single dose of fluconazole eight weeks after treatment (60% *vs.* 35%, *p* < 0.05) [58]. In this study, relapse was defined as reappearance/worsening of clinical symptoms or positive mycology following a negative test. Additionally, a significantly greater mycological cure rate was shown for fluconazole at 8 weeks than itraconazole (65% *vs.* 20%, *p* < 0.05) [58]. While it has been established that a single dose of itraconazole is not ideal, a single dose of fluconazole may be effective treatment for PV.

Dehghan *et al.* (2010) conducted a double-blind, randomized clinical trial comparing a single dose of 400 mg fluconazole to twice daily 1% clotrimazole cream for 14 days [13]. Efficacy was measured as percent lesion clearance, with categories of complete (≥95% lesion clearance), incomplete (50%–95% lesion clearance), and no clinical response (<50% lesion clearance). Four weeks after treatment, the number of patients experiencing complete or incomplete clinical response was significantly greater with clotrimazole cream compared to fluconazole (complete 95% *vs.* 82% and incomplete 19% *vs.* 5%, *p* = 0.044); however, by 12 weeks, complete clinical response was non-significantly higher for the fluconazole group than the clotrimazole group (92% *vs.* 82%) [13]. Recurrence between weeks 4 and 12 or no clinical response at week 12 was observed in 3 patients receiving fluconazole and 10 patients receiving clotrimazole [13]. It is inconclusive if topical clotrimazole is more effective than fluconazole, yet clear that fluconazole 300 mg weekly for 2 weeks and a single 450 mg dose of fluconazole are appropriate for treatment of PV. Patients may find this alternative more attractive than other topical or oral treatments.

### 3.3. Pramiconazole

Pramiconazole is a relatively new triazole that disrupts ergosterol synthesis in fungal cells. It has been shown to be active *in vitro* against dermatophytes, Candida species, and Malassezia species. At concentrations <1 µg/mL, pramiconazole activity was twice that of itraconazole against Candida species, and 10 times greater than ketoconazole against Malassezia species [64]. A Phase II trial of 19 patients with PV evaluated the safety and efficacy of 200 mg pramiconazole daily for 3 days and patients were monitored for 30 days (Day 4, 10, 30) [60]. Across the duration of the study, clinical signs/symptoms (erythema, itching, and desquamation each rated on a five-point scale for a global clinical evaluation) were significantly reduced compared to baseline, *p* < 0.001 [60]. Ten days after the start of treatment, 8 patients were KOH-negative; by 30 days, all 19 patients were KOH-negative. No serious adverse events (AEs) were reported but nine patients (47%) reported AEs, with headache being the most common [60].

Further investigation evaluated five regimens of pramiconazole compared to placebo: 100, 200, or 400 mg single dose of pramiconazole, or 200 mg pramiconazole daily for 2 or 3 days [61]. Patients were evaluated at day 14 and 28 for mycological cure (KOH-negative) and clinical symptoms (erythema, itching, and desquamation each rated on a five-point scale). Complete cure (score of 0 for all clinical symptoms and negative KOH) was significantly higher in the 200 mg single dose (59%), 400 mg single dose (52%), 200 mg for 2 days (72%), and 200 mg for 3 days (85%) compared to the placebo group (16%, *p* = 0.003, *p* = 0.013, *p* < 0.001, *p* < 0.001, respectively) [61]. Similarly, all treatments, with the exception of the 100 mg single dose, produced significantly higher mycological cure than placebo treatment (all groups *p* < 0.001, Table 4). The proportion of patients reporting at least one treatment-emergent AE was not dose dependent and ranged from 31% (100 mg single dose) to 46% (200 mg for 3 days) [61]. Diarrhea and nausea were the most common treatment-emergent AEs, with the study drug formulation (hydroxypropyl-β-cyclodextrin) likely contributing to this [61]. Overall, pramiconazole may be a promising treatment for PV (Table 5); however, it remains to be determined the clinical efficacy of pramiconazole in relation to existing oral antifungals.

## 4. Conclusions

PV is one of the most common cutaneous dermatologic conditions worldwide. As Malassezia species are endogenous to the skin flora, this condition is particularly difficult to eradicate. Preventing recurrence of infections is important going forward. In the meantime, there are a number of topical and oral antifungal treatments that are effective in alleviating clinical symptoms and producing mycological cure. Topical therapy is the first line of treatment for PV and may include selenium sulphide, zinc pyrithione, ketoconazole, and terbinafine [7]. When topical treatment is not feasible or desired, itraconazole and fluconazole are viable options, with pramiconazole a potential new therapy [7,63]. PV will persist if left untreated and high recurrence rates support repeated or maintenance therapy. Patients should be aware that hyper- or hypopigmentation may persist and that it could take months to recover normal skin appearance.

Clinical investigations have demonstrated the clinical efficacy of various topical antifungal medications in treating PV [7,8,9], including topical ketoconazole and terbinafine. Ketoconazole foam is a newer option for treatment and may be favorable to shampoo or cream, as easier application may lead to increased patient compliance [20]. Based on the accumulated evidence, treatment once or twice daily for 14 days with topical ketoconazole cream or foam, and once weekly use of ketoconazole shampoo may be effective treatment for PV, with cream or foam showing long-term efficacy. Similarly, topical terbinafine cream should be applied twice daily for 7 days [63]. Treatment efficacy of topical formulations may be lower in more tropical climates [27]. Recent work demonstrating the efficacy of combination topical treatment [22] may provide an alternative treatment.

It appears that the longer the duration of treatment with topical agents, the more favorable the outcomes. Meanwhile, duration and dose does not influence mycological cure for itraconazole and fluconazole [63]. For effective management of PV with oral antifungal treatment, supported regimens are: 200 mg itraconazole daily for 5 or 7 days, 300 mg fluconazole weekly for 2 weeks, or 200 mg pramiconazole daily for 2 days [63]. A medical panel recommends the use of fluconazole, if possible, over itraconazole due to drug interactions [7]. Systematic reviews and meta-analyses confirm that both topical and oral antifungal therapy is superior to placebo treatment; however, there is not sufficient data to assess the efficacy of one treatment over another [63,65]. In practice, physician experience and patient preferences will dictate which treatment is selected.

The advantage to topical treatments is that they are fast-acting and well tolerated. There is less risk of serious adverse effects and limited drug interactions. This is especially apparent with the history of ketoconazole use, where topical formulations of ketoconazole are the major treatment for PV, yet the risk associated with oral use has led to strict re-labeling. Multiple applications of topical medications may be inconvenient and limit patient compliance, especially in cases of PV where large body areas are affected. In these cases, oral antifungals may be preferable to many patients and the short course of oral treatments may help mediate some of the risk associated with these drugs. Relapse is a widespread concern and a likely possibility. Prophylactic treatment may be necessary to alleviate symptoms, especially in more severe cases. Limited research into the effectiveness of antifungal prophylactic treatment has been conducted. Evidence suggests that monthly itraconazole [52] and selenium sulphide [66] may reduce relapse.
